# Popular Instruction

**Published:** 1874-02

**Authors:** 


### POPULAR INSTRUCTION.

"*Notes on the Teeth, for Popular Reading*;" BY H. L. SAGE, D. D. S.

This is a well written, clear and comprehensive little manual of sxty-five pages, the design of which is to give information to the people about their teeth, and it is well adapted to the purpose.

He who carefully reads it, cannot fail to know what is required for the welfare of his teeth. If the people were educated in this matter up to the plain of this little work, the number of decayed and diseased teeth would very rapidly diminish and the pain and suffering now experienced would dwindle to comparative insignificance. As yet, we have but few of these manuals, and most of those that have been written are good ; each one different from any other, so much so that every dentist should have them *all* for distribution ; for each one contains things that the others do not, and one will be better adapted for certain cases than another. And we suggest that dentists cannot make better investment than in these little works.

